# A new species of *Phrynopus* (Amphibia, Anura, Craugastoridae) from upper montane forests and high Andean grasslands of the Pui Pui Protected Forest in central Peru

**DOI:** 10.3897/zookeys.713.20776

**Published:** 2017-11-02

**Authors:** Edgar Lehr, Rudolf von May, Jiří Moravec, Juan Carlos Cusi

**Affiliations:** 1 Department of Biology, Illinois Wesleyan University, P.O. Box 2900, Bloomington, IL 61701, USA; 2 Museum of Zoology, Department of Ecology and Evolutionary Biology, University of Michigan, 2039 Ruthven Museums Building, 1109 Geddes Ave., Ann Arbor, MI 48109, USA; 3 Museum of Vertebrate Zoology, University of California, Berkeley, 3101 Valley Life Sciences Bldg., Berkeley, CA 94720, USA; 4 Department of Zoology, National Museum, 193 00 Prague 9, Czech Republic; 5 Departamento de Herpetología, Museo de Historia Natural, Universidad Nacional Mayor de San Marcos, Av. Arenales 1256, Jesús María, Lima, Peru

**Keywords:** Andes, montane forest, puna, frogs, DNA barcoding, molecular phylogeny, *Phrynopus inti*, new species, Andes, bosque montano, puna, ranas, códigos de barras de ADN, filogenia molecular, *Phrynopus inti*, especie nueva

## Abstract

We describe a new species of *Phrynopus* from the upper montane forests and high Andean grasslands (puna) of the Pui Pui Protected Forest and its close surroundings (Región Junín, central Peru) and compare it morphologically and genetically with other species of *Phrynopus*.

*Phrynopus inti* **sp. n.** is known from four localities outside and two localities inside the Pui Pui Protected Forest between 3350 and 3890 m a.s.l. Studied specimens of the new species are characterized by a snout-vent length of 27.2–35.2 mm in males (n = 6), and 40.4 mm in a single female, by having the skin on dorsum and flanks smooth with scattered tubercles, venter smooth, by lacking a tympanum, and males without vocal slits and nuptial pads. In life, the dorsum is pale grayish brown with or without dark brown blotches, or dorsum blackish brown with small yellow flecks, throat, chest and venter are pale grayish brown with salmon mottling, groin is pale grayish brown with salmon colored flecks, and the iris is golden orange with fine dark brown reticulations. The new species is morphologically most similar to *Phrynopus kauneorum* and *P. juninensis*. For the latter we describe the coloration in life for a specimen obtained at the type locality. A molecular phylogenetic analysis based on mitochondrial and nuclear DNA sequences inferred that the new species is most closely related to *Phrynopus kauneorum*, *P. miroslawae*, *P. tautzorum*, and an undescribed species distributed at high elevation in Región Pasco, central Peru.

## Abstract (es)

Describimos una nueva especie de *Phrynopus* de los bosques montanos altos y los pajonales altoandinos (Puna) del Bosque de Protección Pui Pui y sus áreas cercanas (Región de Junín, Perú central) y la comparamos morfológica y genéticamente con otras especies de *Phrynopus*. *Phrynopus inti* **sp. n.** es conocido de cuatro localidades fuera y dos localidades dentro del Bosque de Protección Pui Pui entre 3350 y 3890 m s.n.m. La nueva especie se caracteriza por tener una longitud hocico-cloaca de 27.2–35.2 mm en machos (n = 6) y 40.4 mm en una hembra, por tener la piel dorsal y los flancos lisos con tubérculos dispersos, el vientre liso, por carecer de un tímpano, y los machos carecer de hendiduras vocales y almohadillas nupciales. En vida, el dorso es marrón grisáceo pálido con o sin manchas marrón oscuro o el dorso es marrón oscuro con pequeñas manchas amarillas; la garganta, pecho y vientre son marrón grisáceo pálido con motas de color salmón, la ingle es marrón grisácea con manchas de color salmón y el iris es dorado naranja con finas reticulaciones marrón oscuro. La nueva especie es morfológicamente muy similar a *Phrynopus kauneorum* y *P. juninensis*. Para este último, describimos la coloración en vida de un espécimen obtenido en la localidad tipo. Un análisis filogenético molecular basado en secuencias de ADN mitocondrial y nuclear infirió que la nueva especie está más estrechamente relacionada con *Phrynopus kauneorum*, *P. miroslawae*, *P. tautzorum*, y una especie no descrita distribuida en zonas altoandinas de la Región Pasco, Perú central.

## Introduction

**Figure 1.**
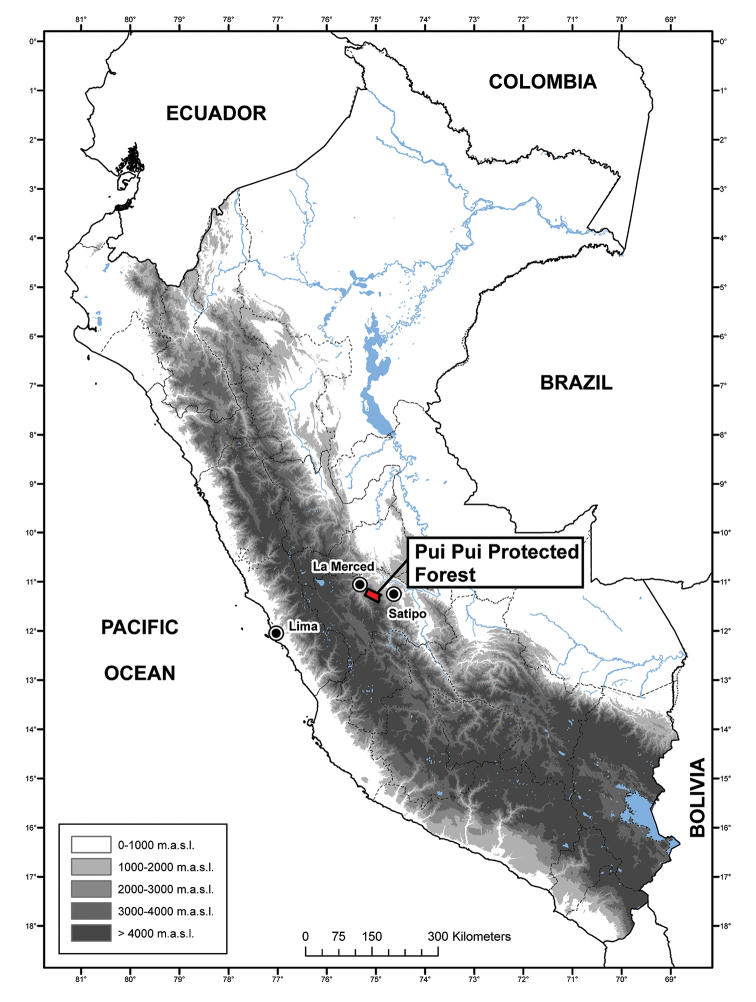
Map of Peru with the Pui Pui Protected Forest indicated in red. Map by J.C. Cusi.

**Figure 2.**
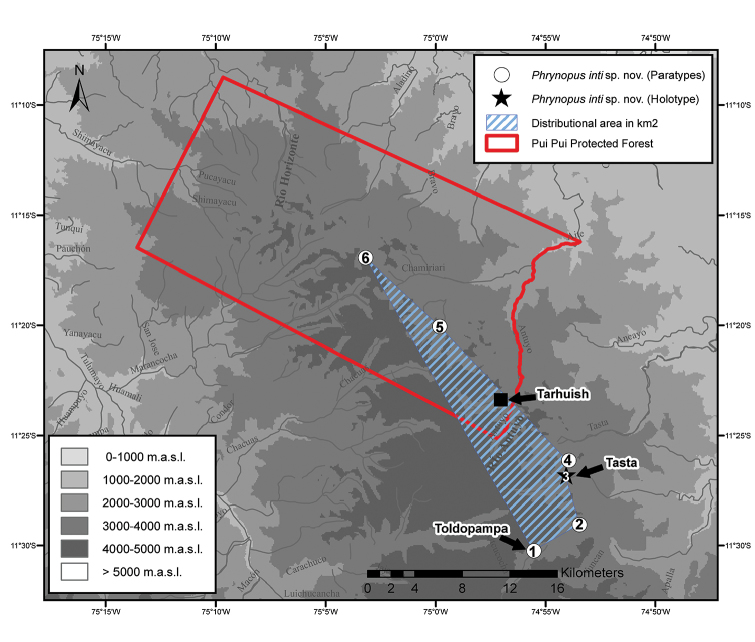
Pui Pui Protected Forest indicated in red outline with collecting sites (1–6) of *Phrynopus inti* **sp. n.**, star indicating type locality, and the estimated distributional area of 101.3 km^2^ in blue. 1 = Toldopampa valley, 3670 m a.s.l., 2 = Satipo-Toldopampa Road at km 134, 3350 m a.s.l., 3 = Quebrada Tasta, 3609 m a.s.l., 4 = *Polylepis* forest patch near trail from Tasta to Tarhuish, 3886 m a.s.l., 5 = Antuyo, 3700 m a.s.l., 6 = close to Laguna Sinchon, 3890 m a.s.l. Map by J.C. Cusi.

The Pui Pui Protected Forest (Bosque de Protección Pui Pui, hereafter PPPF; Figs 1, 2) is located in the Selva Central of Peru and covers 60,000 hectares (30% montane forest, 70% puna habitats) between 1700 and 4500 m a.s.l. (SERNANP 2010). We surveyed the herpetofauna of the PPPF in upper montane forests and high Andean grasslands (puna) between 2012 and 2013 in order to document the amphibian and reptile species richness and to evaluate their conservation status. Among the new amphibians were five new species of *Pristimantis* (Craugastoridae Hedges, Duellman, and Heinicke, 2008) (*P. ashaninka* Lehr & Moravec, 2017; *P. attenboroughi* Lehr & von May, 2017; *P. bounides* Lehr, von May, Moravec, & Cusi, 2017; *P. humboldti* Lehr, von May, Moravec, & Cusi, 2017; and *P. puipui* Lehr, von May, Moravec, & Cusi, 2017) and a new species of *Phrynopus* Peters, 1873. A phylogenetic analysis allowed us to examine the relationships among species of *Phrynopus*, including the new species, and to justify our generic assignment. *Phrynopus* currently contains 34 species (AmphibiaWeb 2017) distributed in montane forests and puna habitats between 2600 and 4490 m a.s.l. in northern and central Peru (Rodríguez and Catenazzi 2017, Duellman and Lehr 2009). Herein we name and describe this new species of *Phrynopus*, supported by morphological and phylogenetic evidence, from upper montane forests and puna habitats.

## Materials and methods

**Fieldwork.** The puna of the PPPF was reached by walking 1.5 days along a trail from Toldopama (11°30'15.4"S, 74°55'32.7"W, 3670 m a.s.l., two hours by car from Satipo) to Tarhuish (11°23'23.2"S; 74°57'02.5"W, 3783 m a.s.l.; Fig. 2) with the help of local guides. Fieldwork was conducted in puna and upper montane forests in 2012 between May 8 and 21 by EL and RvM, and in 2013 between June 21 and July 8 by EL, JM, and JCC. Collected specimens were preserved in 96% ethanol and stored in 70% ethanol.

**Morphological characters.** The format for the description follows Lynch and Duellman (1997), except that the term dentigerous processes of vomers is used instead of vomerine odontophores (Duellman et al. 2006), and diagnostic characters are those of Duellman and Lehr (2009). Taxonomic classification follows Hedges et al. (2008), except that we followed Pyron and Wiens (2011) for family placement. Sex and maturity of specimens were identified by observing gonads through dissections. The senior author measured the following variables to the nearest 0.1 mm with digital calipers under a stereomicroscope: snout-vent length (SVL), tibia length (TL, distance from the knee to the distal end of the tibia), foot length (FL, distance from proximal margin of inner metatarsal tubercle to tip of Toe IV), head length (HL, from angle of jaw to tip of snout), head width (HW, at level of angle of jaw), horizontal eye diameter (ED), interorbital distance (IOD), upper eyelid width (EW), internarial distance (IND), eye-nostril distance (E-N, straight line distance between anterior corner of orbit and posterior margin of external nares), and egg diameter. Fingers and toes are numbered preaxially to postaxially from I–IV and I–V, respectively. We compared the lengths of toes III and V by adpressing both toes against Toe IV; lengths of fingers I and II were compared by adpressing these fingers against each other. All drawings were made using a stereomicroscope and a camera lucida. Photographs of live specimens were used for descriptions of coloration in life and for evaluation of morphological characters that might have been impacted by the preservation process. Information on species for comparative diagnoses was obtained from Duellman and Lehr (2009) and from original species descriptions. For specimens examined see Appendix I. Codes of collections are: **MUSM** – Museo de Historia Natural, Universidad Nacional Mayor de San Marcos, Lima, Peru; **NMP6V** – National Museum, Prague, Czech Republic; **UMMZ** – University of Michigan Museum of Zoology, Ann Arbor, USA. Field number code is: **IWU** – Illinois Wesleyan University, Bloomington, USA. Threat status was evaluated using the IUCN criteria (IUCN Standards an Petitions Subcommittee 2016).

**Maps.** Maps were made with ArcGIS 10.0 (ESRI 2011). The estimated area was calculated by a minimum convex polygon using known sites of occurrence of the species as defined by IUCN (2012).

**Molecular phylogenetic analysis.** Our analysis included DNA sequence data from *Phrynopus* species that were available in GenBank (as of 1 August 2017; Table 1) as well as sequences from other closely related genera (*Lynchius*, *Oreobates*) and more distantly related ones (*Ischnocnema guentheri*, *Hypodactylus brunneus*, and *H. dolops*) as outgroups following the results of Padial et al. (2014). Newly produced sequences include those obtained from seven specimens of the new species and one specimen of *Phrynopus juninensis* collected near Hacienda Cascas, Junín, the type locality of this species (Table 1). Our analysis also included sequences from three mitochondrial and two nuclear genes for several species of *Phrynopus* included in a recent study (De la Riva et al. 2017). The mitochondrial genes were a section of the 16S rRNA gene, a section of the 12S rRNA gene, and the protein-coding gene cytochrome c oxidase subunit I (COI). The nuclear genes were the recombination-activating protein 1 (RAG1) and Tyrosinase precursor (Tyr).

**Table 1.** GenBank accession numbers for the taxa and genes sampled in this study. Bold font indicates new sequences generated for this study. Taxonomy follows Padial et al. (2014).

Taxon	16S	12S	COI	RAG1	Tyr	Voucher_Nbr
*Hypodactylus brunneus*	EF493357	EF493357	na	EF493422	EF493484	KU178258
*Hypodactylus dolops*	EF493394	EF493394	na	EF493414	EF493483	na
*Ischnocnema guentheri*	EF493533	EF493533	na	EF493407	EF493510	na
*Lynchius flavomaculatus*	EU186667	EU186667	na	EU186745	EU186766	KU218210
*Lynchius nebulanastes*	EU186704	EU186704	na	na	na	KU181408
*Lynchius oblitus*	AM039639	AM039707	na	na	na	MTD45954
*Lynchius oblitus*	AM039640	AM039708	na	na	na	MHSNM19914
*Lynchius parkeri*	EU186705	EU186705	na	na	na	KU181307
*Lynchius simmonsi*	JF810004	JF809940	na	JF809915	JF809894	QZ41639
*Oreobates amarakaeri*	JF809996	JF809934	na	JF809913	JF809891	MHNC6975
*Oreobates ayacucho*	JF809970	JF809933	na	JF809912	JF809890	MNCN_IDlR5024
*Oreobates cruralis*	EU186666	EU186666	na	EU186743	EU186764	KU215462
*Oreobates gemcare*	JF809960	JF809930	na	JF809909	na	MHNC6687
*Oreobates granulosus*	EU368897	JF809929	na	JF809908	JF809887	MHNC3396
*Phrynopus auriculatus*	EF493708	EF493708	na	na	na	KU291634
*Phrynopus auriculatus*	MF186348	MF186290	MF186466	na	MF186582	MUBI 6471
*Phrynopus barthlenae*	AM039653	AM039721	na	na	na	SMF81720
*Phrynopus barthlenae*	MF186350	MF186292	MF186464	na	na	MHNSM20609
*Phrynopus bracki*	EF493709	EF493709	na	EF493421	na	USNM286919
*Phrynopus bufoides*	AM039645	AM039713	na	na	na	MHNSM19860
*Phrynopus heimorum*	AM039635	AM039703	MF186462	MF186545	MF186580	MTD45621
*Phrynopus heimorum*	AM039636	AM039704	na	na	na	MTD45622
*Phrynopus horstpauli*	AM039647	AM039715	na	na	na	MTD44334
*Phrynopus horstpauli*	AM039651	AM039719	na	na	na	MTD44333
*Phrynopus horstpauli*	MF186364	MF186303	na	na	MF186584	MTD44335
*Phrynopus inti* **sp. n.**	**MF651901**	na	na	**MF651916**	na	MUSM31203
*Phrynopus inti* **sp. n.**	**MF651902**	**MF651909**	na	**MF651917**	na	MUSM31968
*Phrynopus inti* **sp. n.**	**MF651903**	**MF651910**	na	na	na	MUSM31976
*Phrynopus inti* **sp. n.**	**MF651904**	**MF651911**	na	na	na	MUSM31984
*Phrynopus inti* **sp. n.**	**MF651905**	**MF651912**	na	na	na	NMP6V75584
*Phrynopus inti* **sp. n.**	**MF651906**	**MF651913**	na	**MF651918**	**MF651921**	UMMZ_245218
*Phrynopus inti* **sp. n.**	**MF651907**	**MF651914**	na	**MF651919**	na	UMMZ_245219
*Phrynopus juninensis*	**MF651908**	**MF651915**	na	**MF651920**	na	MUSM33258
*Phrynopus kauneorum*	AM039650	AM039718	na	na	na	MTD44332
*Phrynopus kauneorum*	AM039655	AM039723	na	na	na	MHNSM20595
*Phrynopus miroslawae*	MF186393	MF186312	MF186463	MF186542	MF186585	MUBI 6469
*Phrynopus nicoleae*	MF186394	MF186313	MF186468	MF186546	MF186577	MUBI 6441
*Phrynopus pesantesi*	AM039656	AM039724	na	na	na	MTD45072
*Phrynopus* sp.	AM039657	AM039725	na	na	na	MTD45075
*Phrynopus* sp.	AM039660	AM039728	na	na	na	MTD44759
*Phrynopus tautzorum*	AM039652	AM039720	na	na	na	MHNSM20613
*Phrynopus tribulosus*	EU186725	EU186707	na	na	na	KU291630
*Phrynopus tribulosus*	MF186423	MF186329	MF186469	na	MF186578	MUBI 6451
*Phrynopus tribulosus*	MF186424	MF186330	MF186467	MF186547	MF186579	MUBI 7166

Extraction, amplification, and sequencing of DNA followed protocols previously used for Neotropical terrestrial breeding frogs (Lehr et al. 2005, Hedges et al. 2008). Primers used are listed in Appendix II. We employed the following thermocycling conditions to amplify DNA from each gene using the polymerase chain reaction (PCR). For 16S, we used: 1 cycle of 96 °C/3 min; 35 cycles of 95 °C/30 s, 55 °C/45 s, 72°C/1.5 min; 1 cycle 72°C/7 min. For 12S, we used: 1 cycle of 94°C/1.5 min; 35 cycles of 94°C/45 s, 50°C/1 min., 74°C/2 min; 1 cycle 72°C/10 min. For RAG1, we used: 1 cycle of 96°C/2 min; 40 cycles of 94°C/30 s, 52°C/30 s, 72°C/1.5 min; 1 cycle 72°C/7 min. For Tyr, we used: 1 cycle of 94°C/5 min; 40 cycles of 94°C/30 s, 54°C/30 s, 72°C/1 min; 1 cycle 72°C/7 min. We completed the cycle sequencing reactions by using the corresponding PCR primers and the BigDye Terminator 3.1 (Applied Biosystems), and obtained sequence data by running the purified reaction products in an ABI 3730 Sequence Analyzer (Applied Biosystems). We deposited the newly obtained sequences in GenBank (Table 1).

We used Geneious R6, version 6.1.8 (Biomatters 2013; http://www.geneious.com/) to align the sequences with the built-in multiple alignment program. Prior to conducting phylogenetic analysis, we used PartitionFinder, version 1.1.1 (Lanfear et al. 2012) to select the appropriate models of nucleotide evolution and used the Bayesian information criterion (BIC) to determine the best partitioning scheme and substitution model for each gene. The best partitioning scheme included five subsets (BIC value: 27719.16). The first partition subset included both the 12S and 16S sequences and the best fitting substitution model was GTR+I+G. The remaining four subsets were partitioned according to codon positions as follows (substitution model in parenthesis): one set including the 1^st^ codon position of COI and the 3^rd^ codon position of both RAG1 and Tyr (K80+G); one set with only the 2^nd^ codon position of COI (HKY); one set with only the 3^rd^ codon position of COI (HKY); one set including the 1^st^ and 2^nd^ codon position of RAG1 and the 1^st^ and 2^nd^ codon position of Tyr (HKY+I).

We employed a Bayesian approach using MrBayes, version 3.2.0 (Ronquist and Huelsenbeck 2003) to infer a molecular phylogeny. Our analysis included 44 terminals and a 2684-bp concatenated partitioned dataset. We performed an MCMC Bayesian analysis that consisted of two simultaneous runs of 8 million generations, and we set the sampling rate to be once every 1000 generations. Each run had three heated chains and one “cold” chain, and the burn-in was set to discard the first 25% samples from the cold chain. At the end of the run, the average standard deviation of split frequencies was 0.002257. Following the completion of the analysis, we used Tracer 1.6 (Rambaut and Drummond 2003) to verify convergence. Subsequently, we used FigTree (http://tree.bio.ed.ac.uk/software/figtree/) to visualize the majority-rule consensus tree and the posterior probability values to assess node support. Additionally, we used the R package ‘APE’ (Paradis et al. 2004) to estimate uncorrected p-distances (i.e., the proportion of nucleotide sites at which any two sequences are different).

## Results

**Figure 3.**
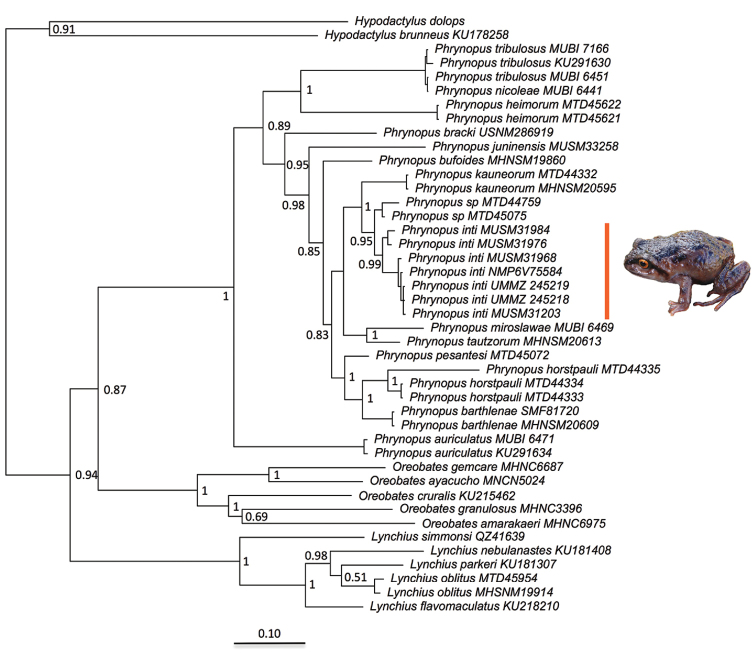
Bayesian maximum clade-credibility tree for species included in this study based on a 2684-bp concatenated partitioned dataset (16S, 12S, COI, RAG1, Tyr) analyzed in MrBayes (posterior probabilities are indicated at each node).

**Molecular phylogenetic analysis.** Placement of the new species in the genus *Phrynopus* was strongly supported by this analysis. We recovered a well-supported tree (Figure 3) that was generally congruent with previous trees (Padial et al. 2014) and supported the unique history of divergence of the new species from other closely related taxa including *Phrynopus kauneorum* Lehr, Aguilar, & Köhler, 2002a, *P. miroslawae* Chaparro, Padial, & De la Riva, 2008, *P. tautzorum* Lehr & Aguilar, 2002, and an undescribed species. Based on the available data, the new species is most closely related to an undescribed species of *Phrynopus* distributed at high elevation (3600–3850 m a.s.l., Lehr et al. 2005) in Región Pasco. This newly identified cryptic species was previously recognized as *P. juninensis* Shreve, 1938 given their similar morphology and coloration (Lehr et al. 2005, Padial et al. 2014) and will be formally named and described in a future paper. The uncorrected p-distances between the new species and all other species of *Phrynopus* ranged between 4.5 and 14.1% (Table 2). The shortest distance occurs between the new species and the undescribed species (uncorrected p-distance 1.5–2.8%) while the uncorrected p-distances between *P. kauneorum* and the new species vary between 3.7 to 4.8% (Table 2). Our analysis also suggests that *P. nicoleae* Chaparro, Padial, & De la Riva, 2008 and *P. tribulosus* Duellman & Hedges, 2008 might represent one species.

**Table 2.** Uncorrected p-distances of the 16S mitochondrial rRNA gene for 30 specimens of *Phrynopus*, including the new species.

		**1**	**2**	**3**	**4**	**5**	**6**	**7**
1	*Phrynopus auriculatus* KU291634	0.000						
2	*Phrynopus auriculatus* MUBI 6471	0.002	0.000					
3	*Phrynopus barthlenae* MHNSM20609	0.138	0.135	0.000				
4	*Phrynopus barthlenae* SMF81720	0.118	0.115	0.000	0.000			
5	*Phrynopus horstpauli* MTD44333	0.114	0.112	0.040	0.039	0.000		
6	*Phrynopus horstpauli* MTD44334	0.114	0.112	0.040	0.039	0.000	0.000	
7	*Phrynopus horstpauli* MTD44335	0.115	0.112	0.040	0.039	0.000	0.000	0.000
8	*Phrynopus pesantesi* MTD45072	0.105	0.103	0.040	0.037	0.035	0.035	0.035
9	*Phrynopus bufoides* MHNSM19860	0.121	0.119	0.073	0.064	0.060	0.060	0.060
10	*Phrynopus tautzorum* MHNSM20613	0.119	0.116	0.077	0.070	0.066	0.066	0.066
11	*Phrynopus miroslawae* MUBI 6469	0.132	0.130	0.084	0.072	0.074	0.074	0.074
**12**	***Phrynopus inti* MUSM31203**	0.125	0.123	0.080	0.070	0.072	0.072	0.073
**13**	***Phrynopus inti*UMMZ 245218**	0.125	0.123	0.080	0.070	0.072	0.072	0.073
**14**	***Phrynopus inti*UMMZ 245219**	0.125	0.123	0.080	0.070	0.072	0.072	0.073
**15**	***Phrynopus inti* MUSM31968**	0.125	0.123	0.080	0.070	0.072	0.072	0.073
**16**	***Phrynopus inti* NMP6V75584**	0.128	0.126	0.082	0.072	0.075	0.075	0.075
**17**	***Phrynopus inti* MUSM31976**	0.123	0.121	0.070	0.064	0.068	0.068	0.069
**18**	***Phrynopus inti* MUSM31984**	0.131	0.128	0.070	0.064	0.066	0.066	0.067
19	*Phrynopus* sp. MTD45075	0.114	0.112	0.069	0.062	0.056	0.056	0.056
20	*Phrynopus* sp. MTD44759	0.119	0.117	0.064	0.059	0.053	0.053	0.053
21	*Phrynopus kauneorum* MHNSM20595	0.128	0.125	0.088	0.079	0.081	0.081	0.082
22	*Phrynopus kauneorum* MTD44332	0.128	0.125	0.088	0.079	0.081	0.081	0.082
23	*Phrynopus bracki* USNM286919	0.110	0.108	0.082	0.074	0.074	0.074	0.075
24	*Phrynopus juninensis* MUSM33258	0.141	0.138	0.126	0.109	0.114	0.114	0.115
25	*Phrynopus heimorum* MTD45621	0.146	0.143	0.137	0.124	0.124	0.124	0.125
26	*Phrynopus heimorum* MTD45622	0.146	0.143	0.137	0.124	0.124	0.124	0.125
27	*Phrynopus nicoleae* MUBI 6441	0.137	0.135	0.124	0.108	0.111	0.111	0.112
28	*Phrynopus tribulosus* KU291630	0.137	0.134	0.124	0.108	0.111	0.111	0.112
29	*Phrynopus tribulosus* MUBI 6451	0.136	0.134	0.124	0.110	0.110	0.110	0.111
30	*Phrynopus tribulosus* MUBI 7166	0.136	0.134	0.124	0.110	0.110	0.110	0.111
		**8**	**9**	**10**	**11**	**12**	**13**	**14**
1	*Phrynopus auriculatus* KU291634							
2	*Phrynopus auriculatus* MUBI 6471							
3	*Phrynopus barthlenae* MHNSM20609							
4	*Phrynopus barthlenae* SMF81720							
5	*Phrynopus horstpauli* MTD44333							
6	*Phrynopus horstpauli* MTD44334							
7	*Phrynopus horstpauli* MTD44335							
8	*Phrynopus pesantesi* MTD45072	0.000						
9	*Phrynopus bufoides* MHNSM19860	0.051	0.000					
10	*Phrynopus tautzorum* MHNSM20613	0.058	0.064	0.000				
11	*Phrynopus miroslawae* MUBI 6469	0.068	0.082	0.049	0.000			
**12**	***Phrynopus inti* MUSM31203**	0.054	0.063	0.066	0.068	0.000		
**13**	***Phrynopus inti*UMMZ 245218**	0.054	0.063	0.066	0.068	0.000	0.000	
**14**	***Phrynopus inti*UMMZ 245219**	0.054	0.063	0.066	0.068	0.000	0.000	0.000
**15**	***Phrynopus inti* MUSM31968**	0.054	0.063	0.066	0.068	0.000	0.000	0.000
**16**	***Phrynopus inti* NMP6V75584**	0.054	0.063	0.070	0.072	0.000	0.000	0.000
**17**	***Phrynopus inti* MUSM31976**	0.048	0.054	0.062	0.064	0.017	0.017	0.017
**18**	***Phrynopus inti* MUSM31984**	0.049	0.054	0.067	0.073	0.023	0.023	0.023
19	*Phrynopus* sp. MTD45075	0.039	0.051	0.049	0.062	0.023	0.023	0.023
20	*Phrynopus* sp. MTD44759	0.042	0.055	0.053	0.065	0.028	0.028	0.028
21	*Phrynopus kauneorum* MHNSM20595	0.052	0.070	0.069	0.077	0.045	0.045	0.045
22	*Phrynopus kauneorum* MTD44332	0.052	0.070	0.069	0.077	0.045	0.045	0.045
23	*Phrynopus bracki* USNM286919	0.068	0.069	0.081	0.075	0.081	0.081	0.081
24	*Phrynopus juninensis* MUSM33258	0.109	0.114	0.117	0.114	0.118	0.118	0.118
25	*Phrynopus heimorum* MTD45621	0.134	0.147	0.136	0.142	0.133	0.133	0.133
26	*Phrynopus heimorum* MTD45622	0.134	0.147	0.136	0.142	0.133	0.133	0.133
27	*Phrynopus nicoleae* MUBI 6441	0.114	0.120	0.136	0.143	0.128	0.128	0.128
28	*Phrynopus tribulosus* KU291630	0.114	0.120	0.136	0.143	0.128	0.128	0.128
29	*Phrynopus tribulosus* MUBI 6451	0.113	0.120	0.136	0.140	0.129	0.129	0.129
30	*Phrynopus tribulosus* MUBI 7166	0.113	0.120	0.136	0.140	0.129	0.129	0.129
		**15**	**16**	**17**	**18**	**19**	**20**	**21**
1	*Phrynopus auriculatus* KU291634							
2	*Phrynopus auriculatus* MUBI 6471							
3	*Phrynopus barthlenae* MHNSM20609							
4	*Phrynopus barthlenae* SMF81720							
5	*Phrynopus horstpauli* MTD44333							
6	*Phrynopus horstpauli* MTD44334							
7	*Phrynopus horstpauli* MTD44335							
8	*Phrynopus pesantesi* MTD45072							
9	*Phrynopus bufoides* MHNSM19860							
10	*Phrynopus tautzorum* MHNSM20613							
11	*Phrynopus miroslawae* MUBI 6469							
**12**	***Phrynopus inti* MUSM31203**							
**13**	***Phrynopus inti*UMMZ 245218**							
**14**	***Phrynopus inti*UMMZ 245219**							
**15**	***Phrynopus inti* MUSM31968**	0.000						
**16**	***Phrynopus inti* NMP6V75584**	0.000	0.000					
**17**	***Phrynopus inti* MUSM31976**	0.017	0.018	0.000				
**18**	***Phrynopus inti* MUSM31984**	0.023	0.023	0.008	0.000			
19	*Phrynopus* sp. MTD45075	0.023	0.024	0.015	0.016	0.000		
20	*Phrynopus* sp. MTD44759	0.028	0.029	0.022	0.017	0.008	0.000	
21	*Phrynopus kauneorum* MHNSM20595	0.045	0.048	0.037	0.040	0.043	0.049	0.000
22	*Phrynopus kauneorum* MTD44332	0.045	0.048	0.037	0.040	0.043	0.049	0.000
23	*Phrynopus bracki* USNM286919	0.081	0.084	0.079	0.083	0.073	0.077	0.086
24	*Phrynopus juninensis* MUSM33258	0.118	0.119	0.113	0.116	0.115	0.116	0.113
25	*Phrynopus heimorum* MTD45621	0.133	0.137	0.141	0.141	0.133	0.135	0.146
26	*Phrynopus heimorum* MTD45622	0.133	0.137	0.141	0.141	0.133	0.135	0.146
27	*Phrynopus nicoleae* MUBI 6441	0.128	0.128	0.130	0.125	0.123	0.119	0.133
28	*Phrynopus tribulosus* KU291630	0.128	0.128	0.130	0.125	0.123	0.119	0.133
29	*Phrynopus tribulosus* MUBI 6451	0.129	0.129	0.132	0.126	0.124	0.120	0.135
30	*Phrynopus tribulosus* MUBI 7166	0.129	0.129	0.132	0.126	0.124	0.120	0.135
		**22**	**23**	**24**	**25**	**26**	**27**	**28**
1	*Phrynopus auriculatus* KU291634							
2	*Phrynopus auriculatus* MUBI 6471							
3	*Phrynopus barthlenae* MHNSM20609							
4	*Phrynopus barthlenae* SMF81720							
5	*Phrynopus horstpauli* MTD44333							
6	*Phrynopus horstpauli* MTD44334							
7	*Phrynopus horstpauli* MTD44335							
8	*Phrynopus pesantesi* MTD45072							
9	*Phrynopus bufoides* MHNSM19860							
10	*Phrynopus tautzorum* MHNSM20613							
11	*Phrynopus miroslawae* MUBI 6469							
**12**	***Phrynopus inti* MUSM31203**							
**13**	***Phrynopus inti*UMMZ 245218**							
**14**	***Phrynopus inti*UMMZ 245219**							
**15**	***Phrynopus inti* MUSM31968**							
**16**	***Phrynopus inti* NMP6V75584**							
**17**	***Phrynopus inti* MUSM31976**							
**18**	***Phrynopus inti* MUSM31984**							
19	*Phrynopus* sp. MTD45075							
20	*Phrynopus* sp. MTD44759							
21	*Phrynopus kauneorum* MHNSM20595							
22	*Phrynopus kauneorum* MTD44332	0.000						
23	*Phrynopus bracki* USNM286919	0.086	0.000					
24	*Phrynopus juninensis* MUSM33258	0.113	0.105	0.000				
25	*Phrynopus heimorum* MTD45621	0.146	0.118	0.113	0.000			
26	*Phrynopus heimorum* MTD45622	0.146	0.118	0.113	0.000	0.000		
27	*Phrynopus nicoleae* MUBI 6441	0.133	0.111	0.121	0.119	0.119	0.000	
28	*Phrynopus tribulosus* KU291630	0.133	0.111	0.121	0.119	0.119	0.000	0.000
29	*Phrynopus tribulosus* MUBI 6451	0.135	0.110	0.123	0.121	0.121	0.002	0.002
30	*Phrynopus tribulosus* MUBI 7166	0.135	0.110	0.123	0.121	0.121	0.002	0.002
		**29**	**30**					
1	*Phrynopus auriculatus* KU291634							
2	*Phrynopus auriculatus* MUBI 6471							
3	*Phrynopus barthlenae* MHNSM20609							
4	*Phrynopus barthlenae* SMF81720							
5	*Phrynopus horstpauli* MTD44333							
6	*Phrynopus horstpauli* MTD44334							
7	*Phrynopus horstpauli* MTD44335							
8	*Phrynopus pesantesi* MTD45072							
9	*Phrynopus bufoides* MHNSM19860							
10	*Phrynopus tautzorum* MHNSM20613							
11	*Phrynopus miroslawae* MUBI 6469							
**12**	***Phrynopus inti* MUSM31203**							
**13**	***Phrynopus inti*UMMZ 245218**							
**14**	***Phrynopus inti*UMMZ 245219**							
**15**	***Phrynopus inti* MUSM31968**							
**16**	***Phrynopus inti* NMP6V75584**							
**17**	***Phrynopus inti* MUSM31976**							
**18**	***Phrynopus inti* MUSM31984**							
19	*Phrynopus* sp. MTD45075							
20	*Phrynopus* sp. MTD44759							
21	*Phrynopus kauneorum* MHNSM20595							
22	*Phrynopus kauneorum* MTD44332							
23	*Phrynopus bracki* USNM286919							
24	*Phrynopus juninensis* MUSM33258							
25	*Phrynopus heimorum* MTD45621							
26	*Phrynopus heimorum* MTD45622							
27	*Phrynopus nicoleae* MUBI 6441							
28	*Phrynopus tribulosus* KU291630							
29	*Phrynopus tribulosus* MUBI 6451	0.000						
30	*Phrynopus tribulosus* MUBI 7166	0.000	0.000					

### Phrynopus inti sp. n.

Taxon classificationAnimaliaAnuraStrabomantidae

http://zoobank.org/C3E88CD6-7AD2-4CFE-8129-A6DB6D747F70

Phrynopus sp. A in Lehr, von May, Moravec, & Cusi (2017)

#### Common name.

English: Inti Andes Frog. Spanish: Rana Andina Inti.

#### Holotype

**Figure 4.**
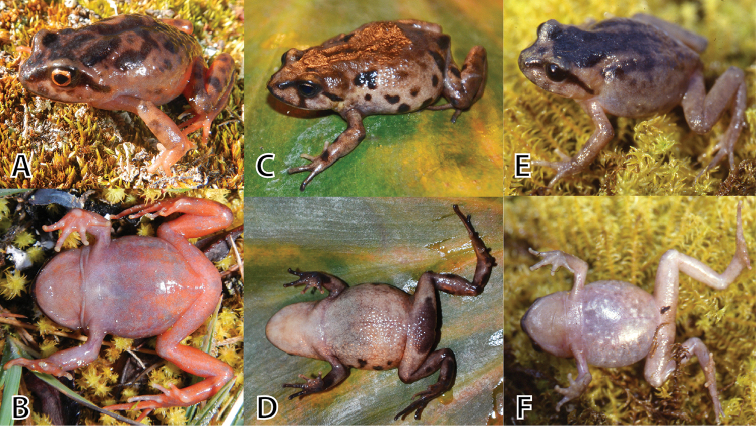
*Phrynopus inti* sp. n. (**A, B** holotype, MUSM 31183, male, SVL 32.5 mm), *P. juninensis* (**C, D** MUSM 33258, female, SVL 33.0 mm), *P. kauneorum* (**E, F** holotype, MUSM 20459, female, SVL 29.1 mm) in dorsolateral and ventral views. Photos by E. Lehr and R. von May (**C, D**).

**Figure 5.**
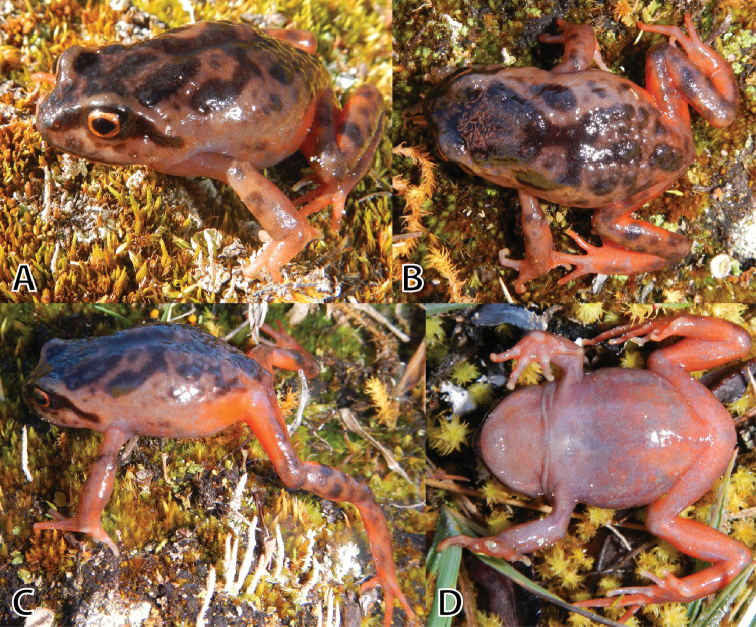
Life male holotype (MUSM 31183, SVL 32.5 mm) of *Phrynopus inti* sp. n. in dorsolateral view (**A**), dorsal view (**B**), flanks, groin, anterior surfaces of thighs (**C**), and ventral view (**D**). Photos by E. Lehr.

**Figure 6.**
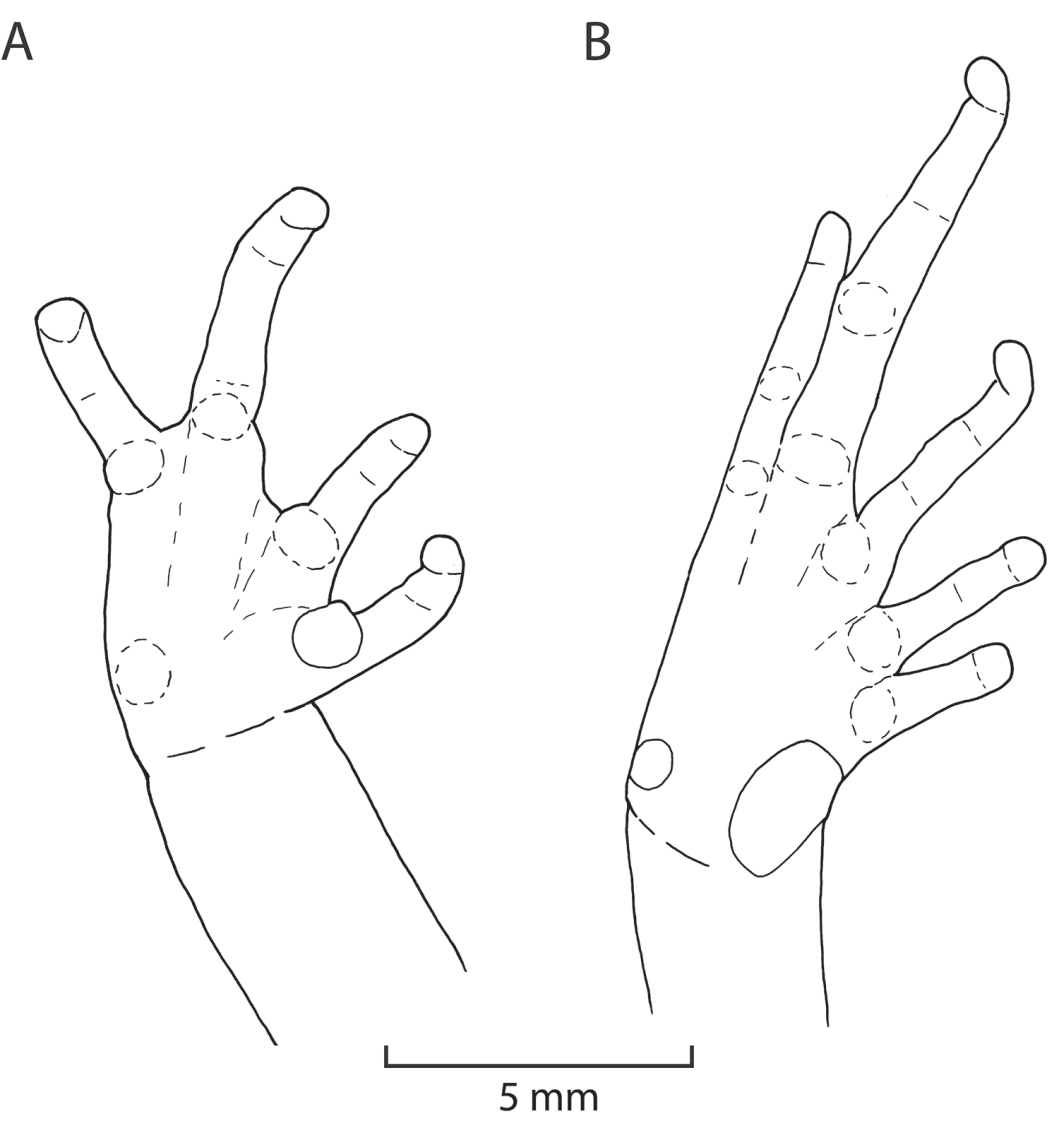
Ventral views of right hand (**A**) and right foot (**B**) of holotype of *Phrynopus inti* sp. n. (MUSM 31183). Drawings by E. Lehr.

(Figs 4A,B, 5, 6). MUSM 31183 (IWU 155), adult male from the buffer zone of the Pui Pui Protected Forest, Quebrada Tasta, forest patch near the house of Evaristo Bórquez Quintana, 11°26'48.8"S, 74°54’2.8"W, 3609 m a.s.l. (Figs 2, 10C), Provincia Satipo, Región Junín, Peru, collected on 9 May 2012 by E. Lehr and R. von May.

#### Paratypes

**Figure 7.**
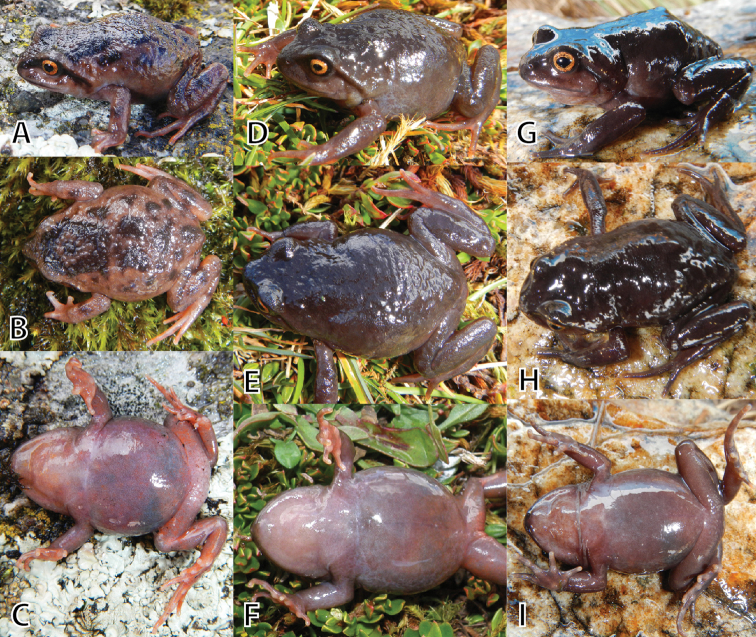
Variation of male paratypes of *Phrynopus inti* **sp. n.** in dorsolateral, dorsal, and ventral views. **A–C** (UMMZ 245220, SVL 27.4 mm), **D–F** (MUSM 31976, SVL 35.2 mm), **G–I** (MSUM 31984, SVL 35.1 mm) . Photos by E. Lehr, and by J.C. Cusi (**E**).

**Figure 8.**
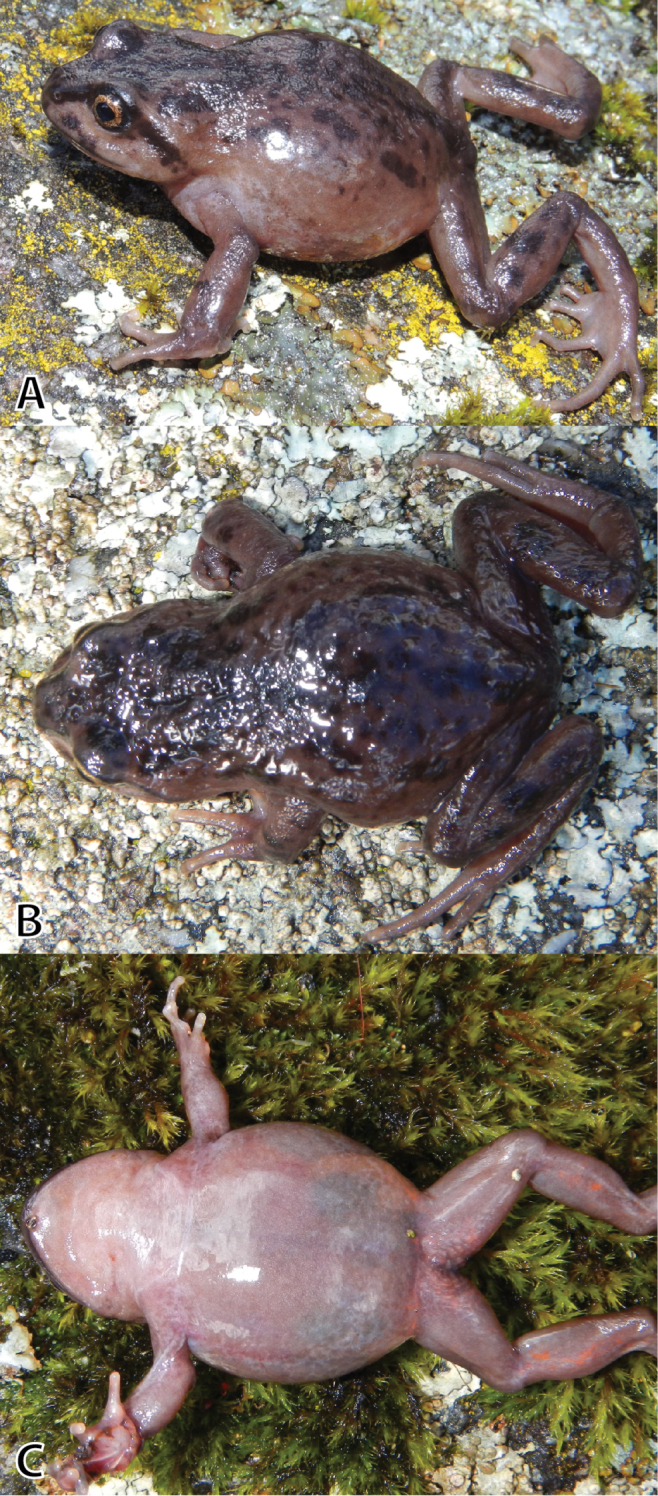
Female paratype of *Phrynopus inti* **sp. n.** (MUSM 31968, SVL 40.4 mm) in dorsolateral (**A**), dorsal (**B**), and ventral views (**C**). Photos by E. Lehr and J. Moravec (A).

**Figure 9.**
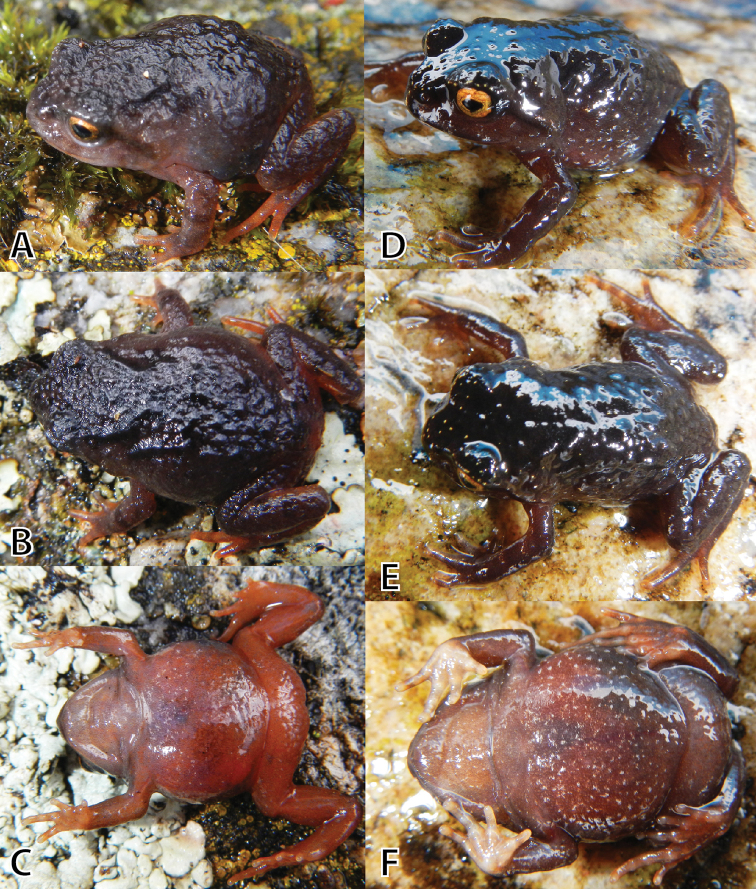
Variation of juvenile paratypes of *Phrynopus inti* sp. n. in dorsolateral, dorsal, and ventral views. **A–C** (MUSM 31969, SVL 16.0 mm), **D–F** (NMP6V 75587, SVL 20.3 mm). Photos by E. Lehr.

(Figs 7, 8, 9). A total of 15, all from Provincia Satipo, Región Junín, Peru (for detailed information see below): 5 males (MUSM 31976, 31984, 31203, NMP6V 75584, UMMZ 245220), 1 female (MUSM 31968), 9 juveniles (MUSM 31184, 31969, 31974, 31985, NMP6V 75585–87, UMMZ 245218, 245219).

MUSM 31184, UMMZ 245218, 245219, collected with the holotype on 9 May 2012 by E. Lehr and R. von May; MUSM 31203, near trail from Tasta to Tarhuish (first cumbre), *Polylepis* forest patch, 11°26'8.6"S, 74°53'56.5"W, 3886 m a.s.l. collected on 20 May 2012 by E. Lehr and R. von May; MUSM 31968, 31969, UMMZ 245220, Toldopampa, 11°30'15"S, 74°55'33"W, 3670 m a.s.l., collected on 22 June 2013 by E. Lehr, J. Moravec, and J.C. Cusi; NMP6V 75584, from Sector Carrizal, Satipo-Toldopampa Road at km 134 on left side of road coming from Satipo, 11°29'03.5"S, 74°53'27.3"W, 3350 m a.s.l., collected on 23 June 2013 by E. Lehr, J.C. Cusi, and J. Moravec; MUSM 31974, 31976, NMP6V 75585, Antuyo, 11°20'03.7"S, 74°59'49.1"W, 3700 m a.s.l., collected on 27 June 2013 by E. Lehr, J.C. Cusi, and J. Moravec; MUSM 31984, 31985, NMP6V 75586, 75587, Laguna Sinchon, 11°16'56.3"S, 75°03'11.7"W, 3890 m, collected on 30 June 2013 by E. Lehr, J.C. Cusi, and J. Moravec.

#### Generic placement.

We assign this species to *Phrynopus* based on molecular evidence (Fig. 3).

#### Diagnosis.

A species of *Phrynopus* having the following combination of characters: (1) Skin on dorsum and flanks shagreen with scattered, low tubercles, more dense on dorsum; skin on venter smooth; discoidal fold absent, thoracic fold present; prominent supratympanic fold; dorsolateral folds absent; (2) tympanic membrane and tympanic annulus absent; (3) snout rounded in dorsal and lateral views; (4) upper eyelid without enlarged tubercles; width of upper eyelid narrower than IOD; cranial crests absent; (5) dentigerous processes of vomers minute or absent; (6) vocal slits and nuptial pads absent; (7) Finger I shorter than Finger II; tips of digits bulbous, rounded; (8) fingers without lateral fringes; (9) ulnar and tarsal tubercles absent; (10) heel without tubercles; inner tarsal fold absent; (11) inner metatarsal tubercle rounded, about three times as large as ovoid outer metatarsal tubercle; supernumerary plantar tubercles absent; (12) toes without lateral fringes; basal webbing absent; Toe V slightly longer than Toe III; toe tips bulbous, rounded, about as large as those on fingers; (13) in life, dorsum pale grayish brown with or without dark brown blotches or blackish brown with small yellow flecks; throat, chest and venter pale grayish brown with salmon mottling, groin pale grayish brown with salmon colored flecks; iris golden orange with fine dark brown reticulations; (14) SVL 27.2–35.2 mm in males (n = 6), and 40.4 mm in single female.

#### Comparisons.

*Phrynopus inti* sp. n. is readily distinguished from its 34 congeners in Peru (AmphibiaWeb 2017), by its relatively large SVL (except for *P. juninensis* and *P. kauneorum*) of up to 40.4 mm, by having the groin pale grayish brown with salmon colored flecks, the venter pale grayish brown with salmon mottling and the iris golden orange with fine dark brown reticulations. *Phrynopus inti* sp. n. is most similar to the large central Peruvian species *P. juninensis* (SVL up to 43.1 mm, Duellman and Lehr 2009) and *P. kauneorum* (SVL up to 56.4 mm, Lehr et al. 2002b), Fig. 4. All three species share a gray ground coloration and dark brown canthal and supratympanic stripes, lack dorsolateral folds and males lack vocal slits and nuptial pads, but can be distinguished as follows: *Phrynopus inti* sp. n. has weak postocular folds (absent in both *P. juninensis* and *P. kauneorum*), has dentigerous processes of vomers (absent in *P. juninensis*, present in *P. kauneorum*), skin on dorsum shagreen with scattered, low tubercles (smooth to weakly areolate in *P. juninensis*, smooth in *P. kauneorum*), skin on venter smooth (areolate in *P. juninensis*, smooth in *P. kauneorum*), dorsum pale grayish brown with or without dark brown blotches or blackish brown with small yellow flecks (dorsum grayish brown with dark brown markings in *P. juninensis*, dorsum pale brown to tan with dark brown markings in *P. kauneorum*), venter pale grayish brown with salmon mottling (pale brown with gray blotches in *P. juninensis*, pinkish to grayish tan in *P. kauneorum*), and the iris is golden orange (copper in *P. juninensis*, dark brown in *P. kauneorum*).

#### Description of the holotype.

Head as wide as body, wider than long, HW 110% of HL; HW 38% of SVL; HL 35% of SVL; snout short, rounded in dorsal and lateral views (Figs 5A, B), ED larger than E–N distance (ED 148% of E–N); nostrils protuberant, directed dorsolaterally; canthus rostralis slightly curved in dorsal view, rounded in profile; loreal region slightly concave; lips rounded; upper eyelid without enlarged tubercles; EW slightly narrower than IOD (EW 94% of IOD); postocular folds low, extending from posterior margin of upper eyelid to level of upper arm insertion (Fig. 5B); supratympanic fold broad, extending from posterior corner of eye to level of upper arm insertion; tympanic membrane and tympanic annulus absent, tympanic region without postrictal tubercles. Choanae small, ovoid, close to but not concealed by palatal shelf of maxilla; dentigerous processes of vomers minute, embedded in mucosa of mouth, widely separated; tongue broad, about twice as long as wide, not notched posteriorly, posterior half free; vocal slits absent.

Skin on dorsum shagreen with scattered, low tubercles, more dense on posterior half of body, dorsolateral folds absent (Fig. 5B); skin on flanks shagreen with few scattered, low tubercles; skin on throat, chest and belly smooth (Fig. 5D); discoidal fold absent, thoracic fold present; cloacal sheath not distinct; cloacal region without tubercles. Outer surface of forearm without tubercles; outer palmar tubercle barely visible, low, ovoid, slightly smaller than ovoid inner palmar tubercle; supernumerary tubercles absent; subarticular tubercles low, ovoid, most prominent on base of fingers; fingers without lateral fringes; Finger I shorter than Finger II; tips of digits rounded, bulbous, lacking circumferential grooves; nuptial pads absent (Fig. 6A).

Hind limbs long and slender, TL 39% of SVL; FL 43% of SVL; dorsal surface of hind limbs shagreen with few low tubercles; anterior surfaces of thighs shagreen, posterior surfaces of thighs weakly areolate; heel without a conical tubercle; outer surface of tarsus without tubercles; outer metatarsal tubercle rounded, weakly conical, about four times as large as prominent ovoid inner metatarsal tubercle; supernumerary plantar tubercles absent; subarticular tubercles low, ovoid in dorsal view, most distinct on base of toes; toes without lateral fringes; basal webbing absent; toe tips bulbous, rounded, lacking circumferential grooves, about as large as those on fingers; relative lengths of toes: 1 < 2 < 3 < 5 < 4; Toe V slightly longer than Toe III (Fig. 6B).

**Measurements of the holotype (in mm).** SVL 32.5; tibia length 12.7; foot length 14.0; head length 11.3; head width 12.5; eye diameter 3.4; interorbital distance 3.5; upper eyelid width 3.3; internarial distance 2.9; eye-nostril distance 2.3.

**Coloration of the holotype in life (Fig. 5).** Dorsum pale grayish brown with dark brown blotches, a dark brown X-shaped marking on shoulder region and an irregular shaped dark brown interorbital blotch. Flanks paler than dorsum with few pale brown flecks. Canthal and supratympanic stripes dark brown. Upper lip with few pale brown flecks. Arms and legs dorsally with few pale and dark brown blotches and flecks. Throat, chest and venter pale grayish brown with salmon mottling, denser on posterior half of belly and thighs. Groin, posterior surfaces of thighs, posterior surfaces of tibias and dorsal surfaces of feet vibrant salmon colored. Iris golden orange with fine dark brown reticulations.

**Coloration of the holotype in preservative.** Dorsum tan with dark brown blotches and dark brown X-shaped marking on shoulder region and an irregular shaped dark brown interorbital blotch. Flanks paler than dorsum, with few pale brown flecks. Canthal and supratympanic stripes dark brown. Upper lip with few pale brown flecks. Arms and legs dorsally tan with few pale and dark brown blotches and flecks. Groin creamy white. Throat, chest and venter creamy white and pale gray mottled. Ventral surfaces of hand and feet creamy white. Iris pale gray.

#### Variation.

All paratypes (Figs 7–9) are similar to the holotype regarding morphology and proportions (Tables 3, 4). Besides differences in SVL (Tables 3, 4), coloration variation in life is notable. Three males (MUSM 31203, UMMZ 245220 (Fig. 7A–C), 285) are similar to the holotype in coloration except for having much less salmon coloration. One male (MUSM 31976, Fig. 7D–F) has the dorsum uniformly grayish brown without dark brown blotches. One male (MUSM 31984, Fig. 7G–I) has the dorsum blackish brown with small yellow flecks. The single female (MSUM 31968, Fig. 8) is similar in coloration to the holotype except for only having few small flecks of salmon in groin, and ventrally on thighs and shanks. The dorsal coloration of the juveniles (Fig. 9) is similar to the adults (dorsum pale grayish brown with dark brown blotches in MUSM 31969 [Fig. 9A, B], 31974, NMP6V 75585, blackish brown with small yellow flecks in NMP6V 75586, 75587 [Fig. 9D, E], uniformly blackish brown in MUSM 31985). The ventral coloration is different in juveniles. One juvenile (MSUM 31969, Fig. 9C) has the venter reddish brown, three juveniles (MUSM 31974, NMP6V 75585, 75587 (Fig. 9F)) have the venter reddish brown and tan mottled.

**Table 3.** Measurements (in mm) of adult type specimens of *Phrynopus inti* sp. n. M = male, F = female. For other abbreviations see materials and methods.

Characters	MUSM	UMMZ	MUSM	NMP6V	MUSM	MUSM	MUSM
	31203	245220	31183	75584	31984	31976	31968
Sex	M	M	M	M	M	M	F
SVL	27.2	27.4	32.5	34.2	35.1	35.2	40.4
TL	10.3	9.9	12.7	14.0	13.3	14.0	15.7
FL	12.2	11.6	14.0	15.4	13.7	13.8	17.1
HL	9.4	9.9	11.3	11.4	11.9	13.0	13.5
HW	10.5	10.6	12.5	12.2	12.6	13.4	14.5
ED	2.4	2.7	3.4	3.1	3.4	3.1	3.5
IOD	3.0	2.8	3.5	3.7	3.0	3.7	3.5
EW	2.5	2.4	3.3	3.2	2.8	3.3	3.4
IND	2.1	2.5	2.9	2.7	2.6	2.9	3.1
E–N	1.9	1.9	2.3	2.1	2.5	2.6	3.0

**Table 4.** Measurements (in mm) and proportions of male type specimens of *Phrynopus inti* sp. n.; ranges followed by means and one standard deviation in parentheses. For abbreviations see materials and methods.

Characters	*Phrynopus inti* sp. n.
	Males (n = 6)
SVL	27.2–35.2 (31.9 ± 3.4)
TL	9.9–14.4 (12.4 ± 1.7)
FL	11.6–15.4 (13.5 ± 1.2)
HL	9.4–13.0 (11.2 ± 1.2)
HW	10.5–13.4 (12.0 ± 1.1)
ED	2.4–3.4 (3.0 ± 0.4)
IOD	2.8–3.7 (3.3 ± 0.4)
EW	2.4–3.3 (2.9 ± 0.4)
IND	2.1–2.9 (2.6 ± 0.3)
E–N	1.9–2.6 (2.2 ± 0.3)
TL/SVL	0.36–0.41
FL/SVL	0.39–0.45
HL/SVL	0.33–0.37
HW/SVL	0.36–0.39
HW/HL	1.00–1.10
E–N/ED	0.68–0.84
EW/IOD	0.83–0.94

#### Etymology.

The species epithet *inti* is derived from the Quechuan noun “Inti”, the Incan sun god. The golden-orange iris reminds us of the sun.

#### Distribution, natural history, and threat status.

*Phrynopus inti* sp. n. is known from four localities outside and two localities inside the Pui Pui Protected Forest between 3350 and 3890 m a.s.l., covering an estimated area of 101.3 km^2^ (Figs 1, 2).

**Figure 10.**
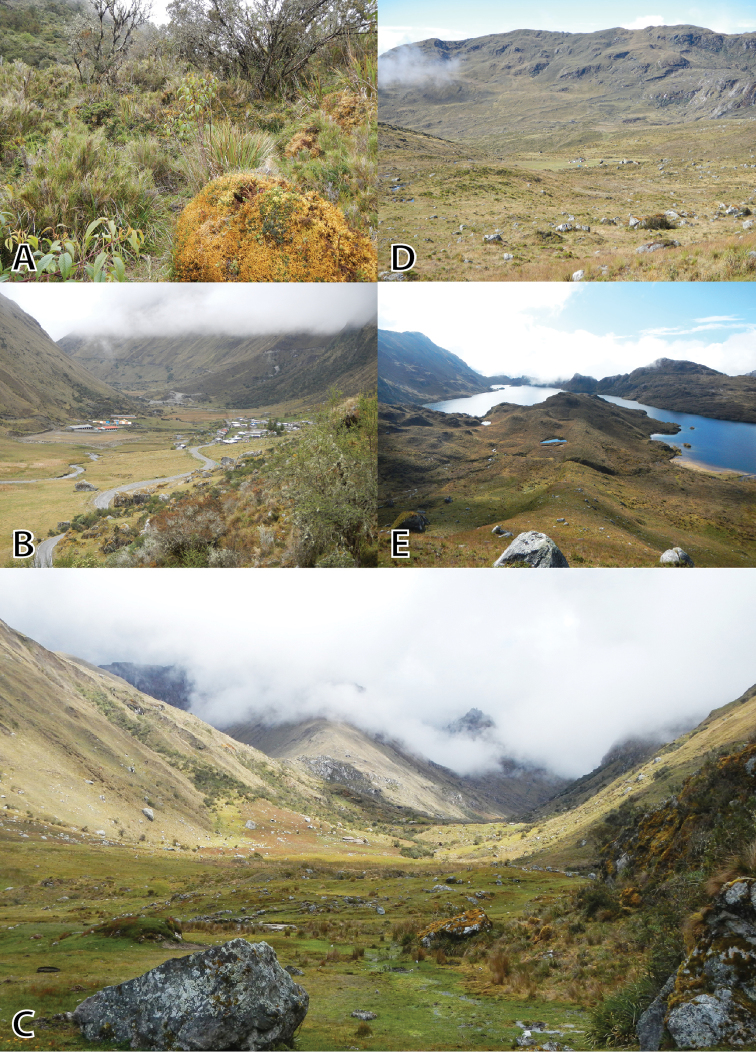
Type locality and habitats of *Phrynopus inti* **sp. n.** Satipo-Toldopampa Road at km 134 on left side of street coming from Satipo, 3350 m a.s.l., 23 June 2013 (**A**); Quebrada Toldopampa, 3670 m a.s.l., 22 June 2013 (**B**); Type locality, Quebrada Tasta, 3609 m a.s.l., 20 May 2012 (**C)**; Antuyo, PPPF, 3700 m a.s.l., 27 June 2013 (**D**); Laguna Sinchon, PPPF, 3890 m a.s.l., 29 June 2013 (**E**). Photos by E. Lehr.

The type locality, Quebrada Tasta (Fig. 2), is outside the PPPF. The holotype and three paratypes (MUSM 31184, UMMZ 245218, 245219) were found in the afternoon in a forest patch under rocks near the house of Evaristo Bórquez Quintana, on 9 May 2012 at 3609 m a.s.l. (Fig. 10C). The vegetation at the type locality consists of *Polylepis* trees, small bushes, ferns, moss, and Peruvian feather grass. No sympatric anurans were recorded. One specimen (MUSM 31203) was found in the afternoon under moss in a *Polylepis* forest patch near the trail from Tasta to Tarhuish at 3886 m a.s.l. Three specimens (MUSM 31968, 31969, UMMZ 245220) were collected in the morning under rocks and in moss in the mountain slopes of the Toldopampa valley close to Toldopampa at 3670 m a.s.l. (Fig. 10B). Specimens were found under rocks and in moss. Sympatric anurans include *Gastrotheca griswoldi* Shreve, 1941. One specimen (NMP6V 75584) was collected under moss in the early afternoon at the Satipo-Toldopampa Road at km 134 on the left side of the road coming from Satipo at 3350 m a.s.l. (Fig. 10A). Sympatric anurans here include *Pristimantis bounides* (MUSM 31970, 31971) and *Gastrotheca griswoldi* (MUSM 31972). Three specimens (MUSM 31974, 31976, NMP6V 75585) were found under rocks and in moss in Antuyo at 3700 m a.s.l. (Fig. 10D). Sympatric anurans here include *Pristimantis attenboroughi* (MUSM 31975) and *Gastrotheca griswoldi* (IWU 290). Four specimens (MUSM 31984, 31985, NMP6V 75586, 75587) were found in the puna in the afternoon in moss close to the Laguna Sinchon at 3890 m a.s.l. (Fig. 10E). Sympatric anurans here include *Pristimantis puipui* (MSUM 31981–83).

One male specimen (MUSM 31203) had as ectoparasites five trombiculid mites on the right side in the area of the upper arm insertion. Such parasites are not uncommon in Andean frogs (e.g., Quinzio and Goldberg 2015, Lehr et al. 2017).

The IUCN Red List criteria (IUCN 2001) consider that if a species occurs in fewer than 10 threat-defined locations and the extent of occurrence (EOO) is < 20,000 km^2^, it should be classified as Vulnerable or Endangered. *Phrynopus inti* sp. n. is known from six localities distributed in the PPPF and its buffer zone (Fig. 10), with an estimated EOO of 101.3 km^2^. As such, this new species might be classified as Vulnerable if we take into account these criteria. However, given that the PPPF may host a greater number of locations (two of them are inside the protected area), we propose that *Phrynopus inti* sp. n. should likely be categorized as Near Threatened (NT). Despite that two locations of the known distribution of *Phrynopus inti* sp. n. are within the PPPF (Fig. 10) and formally protected, other factors such as fungal infections, climate change, pollution, and man-made fires (used to expand grazing areas for livestock) continue to be threats for many Andean amphibians even inside protected areas (Catenazzi and von May 2014). Agriculture and cattle raising are more acute in the Toldopampa valley than in the Tasta valley.

## Discussion

With a snout-vent length of up to 40.4 mm, *Phrynopus inti* sp. n. represents one of the largest species of the genus. Usually, *Phrynopus* species are characterized by a small robust body, short limbs, narrow or only slightly expanded tips of toes and fingers, and absence of a tympanum. These morphological features seem to be associated with a life in moss layers and grass bunches at elevations between 2600 and 4400 m a.s.l. (Rodríguez and Catenazzi 2017, Duellman and Lehr 2009). In the PPPF, however, this niche is widely occupied by several small *Pristimatis* species (*Pristimantis attenboroughi, P. bounides, P. humboldti*, and *P. puipui*), all of which exhibit a similar body form and lifestyle as most species of *Phrynopus*. In particular, two species of *Pristimantis* in the PPPF, *P. attenboroughi, P. puipui*, appear to have adapted to similar niches in upper montane forests and puna that are typically occupied by species of *Phrynopus* with small robust bodies, short limbs, and discs without circumferential groves. Additionally, like in most species of *Phrynopus*, both *P. attenboroughi* and *P. puipui* lack a tympanum. The use of genetic characters in such cases of convergence is necessary to determine the proper generic placement and phylogenetic relationships (Lehr and von May 2017, Lehr et al. 2017). The inclusion of *P. juninensis* from its type locality in our phylogeny (Fig. 2) revealed the existence of a cryptic species (*Phrynopus* sp.) that was previously thought to be *Phrynopus juninensis*. This new species, which is found in an area located >50 km away from the type locality of *P. juninensis*, will be described in the near future.

Our phylogenetic analysis suggested that *Phrynopus nicoleae*, Chaparro, Padial & De la Riva, 2008 is a junior synonym of *Phrynopus tribulosus* Duellman & Hedges, 2008. The high genetic similarity between *P. nicoleae* and *P. tribulosus* was originally identified by De la Riva et al. (2017), who suggested a possible synonymy, but no formal taxonomic action was proposed. Additionally, new evidence suggests that one other species (not included in the tree presented here) is also genetically similar to both *P. nicoleae* and *P. tribulosus* (von May, unpublished). The synonymy among these three species will be discussed in more detail in an upcoming paper.

De la Riva et al. (2017) pointed out an underestimated radiation of craugastorid frogs in the Eastern Andes of Peru and Bolivia and described five new species and a new genus (*Microkayla*). Ten years earlier, De la Riva (2007) described 12 new species from Bolivia and new amphibians are discovered in similar quantities from Andean Peru. The Andes are indeed a hotspot for biodiversity (Myers et al. 2000); five of the six anuran species recorded by us in upper montane and puna habitats of the PPPF represented new species (see Lehr and von May 2017, Lehr et al. 2017 and this paper), and descriptions of other new anuran as well as reptile taxa are expected. Herpetological surveys conducted by us between 2012 and 2014 demonstrate that the PPPF houses unique amphibian assemblages associated with cloud forest and puna habitats. Therefore, the protection of the PPPF and its native flora and fauna in central Andean Peru is of great importance. The beneficial role of any protected area stands out in light of ongoing habitat loss caused by development and land use changes in neighboring areas including the buffer zone surrounding the PPPF.

## Supplementary Material

XML Treatment for Phrynopus inti

## Appendix I

Comparative specimens examined

*Phrynopus barthlenae*: Peru: Huánuco: ca. 15 km SE Maraypata, near Laguna Gwengway, 3680 m: MUSM 20606 (holotype).

*Phrynopus bufoides*: Peru: Pasco: La Victoria, 4100 m: MUSM 18074 (holotype), Paucartambo: Río Gayco, 10°49'9.211"S, 75°58'56.21"W, 4345 m: MUSM 32084.

*Phrynopus curator*: Yanachaga-Chemillén National Park (Sector San Daniel), 3000 m: MUSM 31106 (holotype).

*Phrynopus daemon*: Peru: Huánuco: Distrito de Churubamba, Cordillera de Carpish, Unchog elfin forest, 3341 m: MUSM 32747 (paratype).

*Phrynopus interstinctus*: Peru: Huánuco: Cordillera de Carpish, San Marcos, 3100 m: MUSM 29543 (holotype), 3160 m: MUSM 29544–29545 (paratypes).

*Phrynopus juninensis*: Peru: Junín: road between Cachiyacu and Hacienda Cascas (11°12'43.1"S, 75°35'31.9"W), 3508 m: MUSM 33258.

*Phrynopus kauneorum*: Peru: Huánuco: Chaglla, Palma Pampa, 3020 m: MUSM 20459 (holotype), MUSM 19894, 20700; Huánuco: Carpish de Moyobamba: MUSM 18585.

*Phrynopus peruanus*: Peru: Junín: Puna of Maraynioc (11°21'35.2"S, 75° 28'52.6"W), 3825 m: MHNSM 19977–78.

## Appendix II

**Primers used in this study:**

Locus	Primer		Sequence (5’-3’)	Reference
16S	16SAR	F	CGCCTGTTTATCAAAAACAT	Palumbi et al. (1991)
	16SBR	R	CCGGTCTGAACTCAGATCACGT	Palumbi et al. (1991)
12S	L25195	F	AAACTGGGATTAGATACCCCACTA	Palumbi et al. 1991
	H2916	R	GAGGGTGACGGGCGGTGTGT	Palumbi et al. 1991
COI	dgLCO1490	F	GGTCAACAAATCATAAAGAYATYGG	Meyer et al. (2005)
	dgHCO2198	R	TAAACTTCAGGGT GACCAAARAAYCA	Meyer et al. (2005)
RAG1	R182	F	GCCATAACTGCTGGAGCATYAT	Heinicke et al. (2007)
	R270	R	AGYAGATGTTGCCTGGGTCTTC	Heinicke et al. (2007)
Tyr	Tyr1C	F	GGCAGAGGAWCRTGCCAAGATGT	Bossuyt and Milinkovitch (2000)
	Tyr1G	R	TGCTGGGCRTCTCTCCARTCCCA	Bossuyt and Milinkovitch (2000)
